# Intramyocardial immunomodulation with human CD16^+^ monocytes to treat myocardial infarction in pig: a blind randomized preclinical trial

**DOI:** 10.3389/fcvm.2024.1427023

**Published:** 2024-08-07

**Authors:** Raimondo Ascione, Vito D. Bruno, Tom Johnson, Eva Sammut, Andrew Bond, Daniel Lopez-Baz, Julia Deutsch, Mick Bailey, Amedeo Chiribiri, Ashish Patel, Andrew Baker, Bijan Modarai

**Affiliations:** ^1^ Faculty of Health Science, Bristol Heart Institute and Translational Biomedical Research Centre, University of Bristol, Bristol, United Kingdom; ^2^ Faculty of Health Science, Langford Vets and Translational Biomedical Research Centre, University of Bristol, Bristol, United Kingdom; ^3^ School of Biomedical Engineering and Imaging Sciences, King’s College London, London, United Kingdom; ^4^ Vascular Surgery, King’s College London, London, United Kingdom; ^5^ Centre for Cardiovascular Science, University of Edinburgh, Edinburgh, United Kingdom

**Keywords:** myocardial infarction, ischemic heart failure, treatment, immunomodulation, monocytes, intramyocardial delivery

## Abstract

**Background:**

Human CD16^+^ monocytes (hCD16^+^ Ms) have proangiogenic properties. We assessed the feasibility, safety and efficacy of hCD16^+^ Ms in a porcine model of myocardial infarction (MI).

**Methods and results:**

A total of 27 female Large White pigs underwent MI with reperfusion and cardiac magnetic resonance (CMR). Five days later, animals received intramyocardial injections of hCD16^+^ Ms in saline (*n* = 13) or saline only (*n* = 14). hCD16^+^ Ms were selected from leucocyte cones. Feasibility/safety endpoints included injury at injected sites, malignant arrhythmias, cancer, haematoma, left ventricular (LV) dilatation, troponin release and downstream organ injury. Co-primary efficacy outcome included LV scar and ejection fraction (LVEF) at 30-day post-injections by CMR. Immunohistochemistry included neo-angiogenesis, fibrosis, markers of myofibroblast and inflammation. Four animals were excluded before injections due to untreatable malignant arrhythmias or lung injury. Median cell number and viability were 48.75 million and 87%, respectively. No feasibility/safety concerns were associated with the use of hCD16^+^ Ms. The LV scar dropped by 14.5gr (from 25.45 ± 8.24 to 10.8 ± 3.4 gr; −55%) and 6.4gr (from 18.83 ± 5.06 to 12.4 ± 3.9gr; −30%) in the hCD16^+^ Ms and control groups, respectively (*p* = 0.015). The 30-day LVEF did not differ between groups, but a prespecified sub-analysis within the hCD16^+^ Ms group showed that LVEF was 2.8% higher and LV scar 1.9gr lower in the subgroup receiving a higher cell dose. Higher tissue levels of neo-angiogenesis, myofibroblast and IL-6 and lower levels of TGF-β were observed in the hCD16^+^ Ms group.

**Conclusions:**

The use of hCD16^+^ Ms in acute MI is feasible, safe and associated with reduced LV scar size, increased tissue levels of neo-angiogenesis, myofibroblasts and IL-6 and reduced pro-fibrotic TGF-β at 30-day post-injections. A higher cell dose might increase the LVEF effect while reducing scar size, but this warrants validation in future studies.

## Introduction

The management of >26 million global patients developing chronic heart failure (CHF) after myocardial infarction (MI) (1, 2) remains a major global challenge for healthcare providers. This condition remains largely irreversible making new therapeutic approaches desperately needed (3). MI leads to a marked early loss of cardiomyocytes, while an additional number of vulnerable cardiomyocytes is lost in the border zone (BZ) of the infarct over several days/weeks after the acute event due to persisting ischemia (3, 4). Therapeutic approaches able to prevent this early or subacute loss of cardiomyocytes might mitigate the risk of developing CHF.

Endothelial progenitors, mesenchymal stem cells, bone marrow-derived mononuclear cells and cardiac stem cells have been trialled in patients over the last 15 years (4). These cell therapies have often shown promising results in small rodents (5) but have invariably failed to show meaningful clinical effects when translated into large animals or patients (6) due to poorly relevant models, ill-defined cell populations, lack of scaling up, suboptimal methods of cell delivery and/or exclusive targeting of cardio-myogenesis with less focus on neo-angiogenesis and immunomodulation (5, 6).

The immune response post-MI can be divided into early proinflammatory phase and late inflammatory resolution or reparative phase, involving components of both the innate and adaptive immune systems (7). Harnessing of the immune responses has been associated with enhanced cardiac repair (8). In addition, cardiac healing has been associated with the type and extent of immune responses to tissue injury (8–11), leading to better recovery after preclinical MI or better clinical prognosis in patients (8, 12, 13). The myocardial immune response following MI involves the recruitment of consecutive waves of circulating monocytes (13–15), which, in response to the microenvironment, can differentiate into macrophages, dendritic cells and/or other cell types (16). Hence, monocytes and derived cells may contribute to tissue healing/repair following MI by impacting inflammation, phagocytosis, proteolysis, angiogenesis, left ventricular (LV) scarring and remodelling (17–19). We have shown that subsets of human circulating monocytes expressing CD16 (hCD16^+^ Ms) have proangiogenic activity when delivered into ischemic tissues (20), that circulating levels are 10-fold higher in patients with critical limb ischemia compared to healthy donors and that resolution of ischemia is associated with normalization of circulating levels (20). Others have also suggested that CD16^+^ Ms may trigger neo-angiogenesis (21). These findings indicate that increasing tissue levels of hCD16^+^ Ms might have a therapeutic effect.

Mechanistically, CD16^+^ monocytes are known to express high levels of CX3CR1 and low levels of chemokine receptors in the infarcted myocardium, where they become the predominant monocyte subtype 3–5 days post-MI (22). They have been associated with myocardial reparative potential by releasing anti-inflammatory cytokines and growth factors to stimulate cell proliferation, angiogenesis and ECM production (23, 24).

This preclinical trial aimed to ascertain the feasibility, safety and preliminary reparative efficacy of direct intramyocardial hCD16^+^ Ms delivery in a porcine model of closed chest MI with reperfusion under immunosuppression.

## Materials and methods

An extended version of Materials and methods is shown in the Supplementary File. The data underlying this article are available in the article and its online Supplementary Material. Full raw data, statistical methods and trial materials can be made available on request.

### Ethics

Human CD16^+^ monocytes (hCD16^+^ Ms) were selected from leucocyte cones produced as a by-product of the apheresis process of blood donated to the National Health Service Blood and Transplant (NHSBT). Ethical approval was obtained from the South East London Research Ethics Committee approval (Ref. 10/H0804/67). Donors gave generic consent for research use as part of the donation process and are not identifiable. The tests undertaken on the human tissue conformed to the principles outlined in the Declaration of Helsinki. The *in vivo* animal-regulated procedures were conducted in line with UK Home Office regulations (Animal Act 1986) at the MHRA-compliant Translational Biomedical Research Centre (TBRC) (Bristol, UK). The procedures were conform to the guidelines from Directive 2010/63/EU and were undertaken under project licenses (7008975 and PP4585512) granted by the Home Office after formal review and approval by the University of Bristol Animal Welfare and Ethics Review Body (AWERB). The report of research data from animal experiments is in keeping with the ARRIVE (Animals in Research: Reporting *In Vivo* Experiments) guidelines (25).

### Human Cd16^+^ Ms selection

The hCD16^+^ Ms product was manufactured via immunomagnetic selection under GMP-compliant conditions at King's College London (KCL), according to a strict SOP (Supplementary Figure S1). Final product characterization included cell count, purity and viability. The final product was suspended in N/Saline to achieve a final volume of 3 ml, packed in cold bags, stored overnight and couriered early next morning from KCL to TBRC Bristol for immediate injection. At TBRC, prepacked saline with or without cells was handled at clinical standards and drawn in opaque syringes just before being handled to the surgeon for blind injection for direct intramyocardial injections under direct vision (within 24 h from product manufacturing).

### Porcine MI procedure and delivery of hCD16^+^ Ms

**Figure 1:**
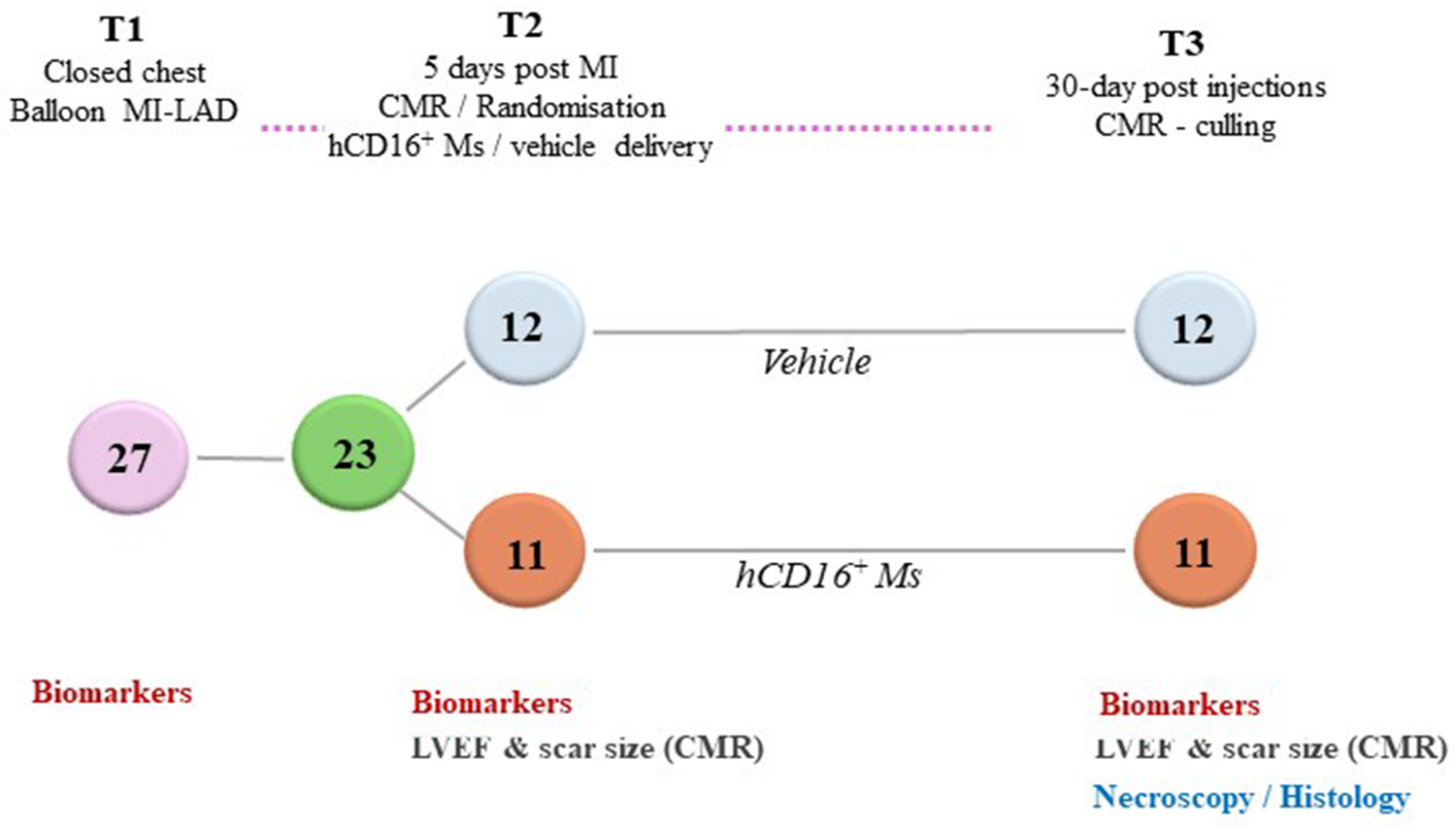
Schematic of porcine trial overtime: MI, myocardial infarction; LAD, left anterior descending artery; CMR, cardiac magnetic resonance; hCD16^+^ MS, hCD16^+^ monocytes; LVEF, left ventricular ejection fraction.

Female Large White swine were used (weight, 55–60 kg). The MI procedure was as previously reported (26) (Figure 1; Supplementary File). Briefly, aspirin 300 mg was administered daily with food from 5 days before MI and until termination. On the MI day, under general anaesthesia (GA), full monitoring and IV heparinization to achieve an activated clotting time (ACT) >300 s, animals were subjected to percutaneous balloon MI procedure of the antero-apical LV territory under fluoroscopic guidance. Amiodarone infusion (300 mg over 90 min) was started before coronary occlusion to prevent ventricular fibrillation (VF). Additional amiodarone bolus (150 mg) and DC cardioversion were used if necessary to treat VF during ischemia. Animals were recovered, and vital parameters were monitored for 45–60 min before return to the maintenance area. Daily clinical grade immunosuppression was started 3 days after MI and included cyclosporine 15 mg/kg/day + methylprednisolone 2 mg/kg/day till termination. *In vivo* characterization of MI scar size and LV function occurred 5 days after MI under GA via baseline cardiac magnetic resonance (CMR) imaging. Next, animals were randomized according to a predefined sequence to receive blindly either hCD16^+^ Ms suspended in saline (hCD16^+^ Ms group) or saline alone (control group) via 10 microinjections (0.3 ml each) targeting LV scar and border zone of the infarcted myocardial territory via mini-thoracotomy according to a predefined LV 17-segment model (Supplementary Figure S1). The rationale of waiting 5 days before injecting cells/placebo after MI was based on hCD16^+^ Ms being reported as the predominant subtype at 3–5 days post-MI (22) as well as obtaining CMR imaging with less tissue oedema, enhancing cell survival by avoiding delivery too close to the MI ischemic event (27) and enhancing the feasibility of future translational in humans where mini-thoracotomy and direct injections are regarded not safe if undertaken too early after MI. On completion, animals were recovered as per routine [(27); Supplementary File]. Thirty days after injections, animals underwent a final GA, repeat CMR followed by termination and tissue sampling.

### CMR image acquisition and analysis

A 3 T scanner with a 32-element phased-array cardiac coil was used (Siemens Prisma, Germany). CMR protocol included cine sequences in long and short axis, pre-contrast T2-STIR and T2 mapping, pre-contrast (native) and post-contrast T1 mapping and extracellular volume (ECV) measurements and early and late gadolinium enhancement (26). Image acquisition was performed by a blind expert radiographer. CMR data interpretation/reporting was performed by two blinded senior radiology investigators using dedicated software (Argus, Siemens AG, Erlangen, Germany).

### Outcome measures

#### Feasibility and safety

Feasibility outcome measures related to the hCD16^+^ Ms product manufacturing in London included obtaining a median cell count >40 million, cell viability >80% and intramyocardial delivery within 24 h at TBRC Bristol. Porcine MI procedural feasibility included obtaining an average LV scar size >10gr at CMR, safe intramyocardial delivery of hCD16^+^ Ms /placebo and 100% survival to the predefined endpoint of all injected animals. Safety outcome measures included serial vital observations, animal well-being and weight gain overtime, full blood count, myocardial injury (serial troponin release) and circulating and tissue-based markers of inflammation (interleukin-6-IL-6). In addition, they included CMR and macroscopy safety indices [LV intramural growth/cancer, excessive LV scarring, infection/abscess, adverse LV remodelling/heart failure—LV end-diastolic diameter (LVEDD) and intramural haematoma/thrombosis]. LV scar and LVEDD were reported as continuous variables while LV intramural growth/cancer, abscess or haematoma/thrombosis were reported as either present or absent. Heart rhythm and malignant ventricular arrhythmias were studied via serial electrocardiogram.

#### *In vivo* efficacy measures

Co-primary CMR measures included LV global scar size and LV ejection fraction at 30 days after injections vs. 5 days after MI before injections. Additional CMR outcome measures included microvascular obstruction (MVO) and global LV volumes.

#### Blood and tissue outcome measures

**Figure 2:**
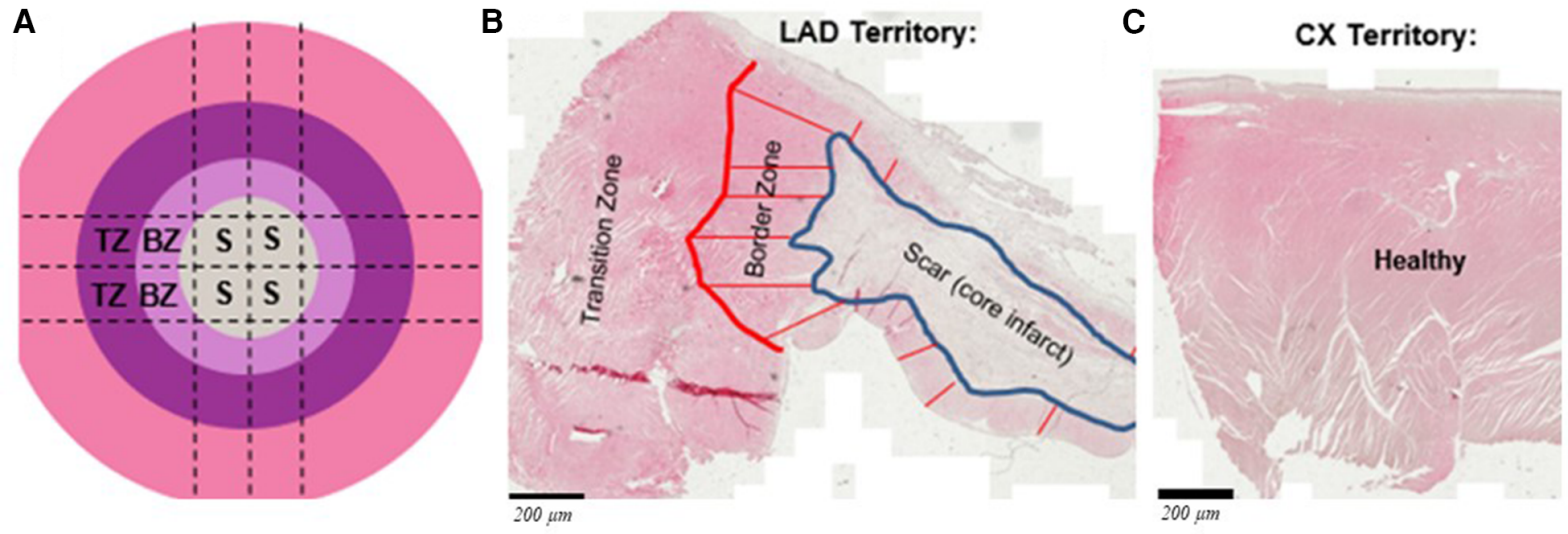
(**A**) Schematic showing the left anterior descending (LAD) artery territory divided for histology and mRNA expression: S, BZ and TZ represent scar, border zone and transition zone, respectively; representative H&E stained tissue sections show the defined zones within the infracted territory (**B**) and healthy from circumplex (Cx) artery territory (**C**). Scale bars are 200 μm.

Blood-based outcomes included serial full blood count, myocardial injury (troponin) and inflammation (IL-6) at baseline (before MI and before the start of immunosuppression), 5 days post-MI (30 min before injections and 1 h after) and 30 days after injections. Histology-based outcomes included histochemical staining and/or mRNA expression for neo-angiogenesis, fibrosis, myofibroblasts, cardiomyocyte function and inflammation (*n* = 8 in each group). Following culling, the infarcted left anterior descending (LAD) territory was collected for each heart along with samples of non-infarcted myocardium from the circumflex artery (CX) territory. Samples for analysis were categorized as scar (S), border zone (BZ) or transition zone (TZ) (Figure 2A) from the LAD territory or as healthy (H) from the CX territory (Figure 2C). Some aliquots were immediately tissue snap frozen in liquid nitrogen and stored at −80°C while others were fixed in 10% formalin before paraffin embedding. Serial sections were cut at 5 µm thickness. Haematoxylin and eosin (H&E) staining of a section of each heart was used to classify regions into S, BZ (the region extending 2 mm around the infarcted area) and TZ (tissue >2 mm away from the edge of S on the tissue section) (Figure 2B). Each within-infarct territory (S, BZ or TZ) was compared alone or cumulatively vs. H territories within the same heart and across groups.

### Macroscopy, myocardial histology staining and mRNA expression

This is fully shown in the Supplementary File. After termination, a blind macroscopy of the whole heart was carried out to re-assess the presence/absence of LV intramural growth/cancer, infection/abscess and/or intramural haematoma/thrombosis; the presence of any of these abnormalities would be investigated by ad-hoc histology. All myocardial samples were stained for markers of neo-angiogenesis (anti-CD31/PECAM-1; platelet endothelial cell adhesion molecule), fibrosis (picrosirius red for collagen), myofibroblasts (anti-vimentin and anti-CD90 (Thy1) and functional cardiomyocytes (anti-desmin). Expanded staining methods and the antibodies used can be found in the Supplementary File. All stained tissue sections were imaged with a Precipoint O8 Slidescanner using a 20× objective, with the exception of CD31 stained tissue that was imaged at higher magnification (40×). To quantify neo-angiogenesis, anti-CD31 (a marker of endothelial cells) was assessed across an average of five images per heart region. Manual counting of capillaries (CD31^+^ staining with one associated nuclei) and arterioles (CD31^+^ with two or more nuclei; Supplementary Figure S1) was undertaken, and data were reported as number of vessels/mm^2^ tissue. The remaining histological analysis was performed on data from three myocardial regions per area, and the percentage of staining per tissue area was determined using colour deconvolution in Fiji ImageJ (Java 1.8.0 64-bit). For mRNA expression 384-well quantitative PCR was used for markers of myocardial fibrosis (TGF-β, CTGF, MMP2, Col1a1, Col1a2, Col3a1), myofibroblasts (CD90, ACTA2, POSTN), cardiomyocyte function (GLUT1, GLUT4) and inflammation (TNFα, IL-1B, IL-6). All mRNA levels were normalized to the housekeeping genes GUSB, PPIA and GAPDH. Further methods and primer sequences are shown in the Supplementary File. Expression was calculated using the 2^–ddCt method.

### Statistical analysis and sample size

Non-parametric analysis was performed by blind statisticians. Numerical clinical and *in vivo* imaging variables are presented as medians and confidence intervals or as mean and standard deviation. The categorical variables are reported as count and percentages. Normality was assessed using the Shapiro–Wilk test. LVEF, scar size and LV volumes measured before cell/placebo injections and at 30 days were compared with the baseline data using a Kruskall–Wallis test. Two-group comparison analysis was performed by Student *t*-test or Mann–Whitney test if data were not normally distributed. Comparison analysis between categorical variables was performed by chi-square test or Fisher exact test as appropriate. A *p*-value of <0.05 was considered statistically significant. One-way ANOVA was used for the initial assessment with Gabriel's test to find differences between pairs of means. Correlations between LVEF and scar size and cell viability and cell count split by medians were calculated as predefined sub-analyses by regression analysis within the hCD16^+^ Ms group. Statistical analyses were performed in IBM SPSS (IBM Corp. Released 2015) and R version 4.1.2 [R Core Team (2021). R: A language and environment for statistical computing. R Foundation for Statistical Computing, Vienna, Austria. URL https://www.R-project.org/]. For mRNA comparison, expression in each region (S, BZ, TZ) within the infarcted LAD territory was compared as fold-change vs. expression in healthy CX regions of each heart. Additional analysis was performed comparing averaged/combined expression across S, BZ and TZ of infarcted LAD territory vs. healthy CX territory. For histology and mRNA comparisons, two-way ANOVA was used to test across hCD16^+^ Ms vs. control groups and regions, while a *post hoc* Tukey HSD test was carried out to observe differences between individual regions. CMR and macroscopy-based evaluation of predefined safety measures were classified either as present or absent.

### Sample size and power calculation

Measuring *in vivo* imaging and clinical outcomes longitudinally via CMR imaging at baseline, just before cell/placebo injections and 4 weeks later in the same animal increases the relative efficiency of the study by more than 80%, assuming a correlation of 0.7 between baseline and final measurements. All calculations are based on cell-specific trials with a 1:1 randomization schedule, using ANCOVA analyses. The co-primary outcomes included LV scar size and LVEF. Power analysis: *LV scar size*, power at 2p = 0.05 with 11 animals in each arm to identify a 5.6 gr (FWHM method) difference between groups; *LVEF*, assumed standard deviation of 7.2% between measurements before cell/placebo injection and 30-day post-injection: 11 animals in each group required to have 80% power at 2p = 0.05. Hence, to allow for excess experimental failure, we included a total sample size of 27. For histology and mRNA comparisons, eight hearts were selected randomly and assessed in each group.

## Results

**Figure 3:**
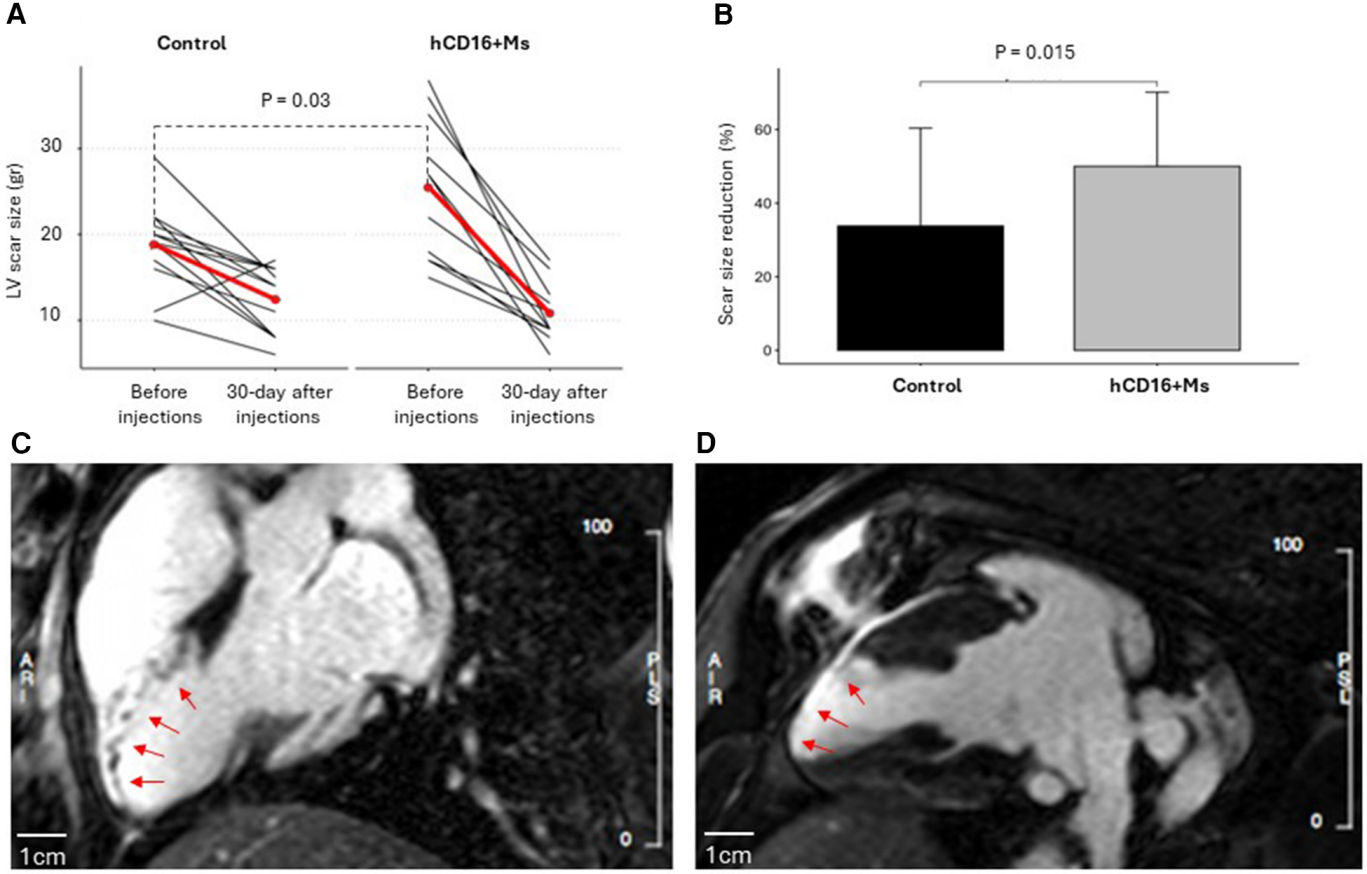
(**A**) LV scar size before infections and 30 days after injections in the control and hCD16+Ms groups: (**B**) percentage reduction in LV scar size in each group at 30-day time point vs. before infections time point; (**C**,**D**) representative experiment from the hCD16^+^ Ms group showing CMR left ventricular late gadolinium enhancement in white representing scar size and distribution in the infarcted territory before injections (**C**) and at 30 days after injections (**D**). Scale bars are 1cm.

Twenty-seven female Large White pigs (mean weight, 63.15, SD 2.93) were subjected to MI. Three animals suffered untreatable ventricular fibrillation (VF) during acute ischemia while one animal suffered lung injury during mini-thoracotomy time point before injections. These four animals were excluded in line with the study protocol. Hence, a total of 23/27 animals were recruited in the trial and underwent injections according to a predefined randomization sequence (*n* = 11 in the hCD16^+^ Ms group and *n* = 12 in the control group). Baseline characteristics including CMR outcome measures are shown in Table 1 and Supplementary Table S1. By chance, the MI scar was larger in the hCD16^+^ Ms group despite randomization (CMR scar size before injections was 25.4 ± 8.24gr vs. 18.8 ± 5.1gr in the hCD16^+^ Ms and control groups respectively; *p* = 0.03; Table 1; Figure 3A). LVEF pre-injections were 45.5 ± 6.3% and 44.3 ± 5.5% in the hCD16^+^ Ms and control groups, respectively (*p* = 0.61). LV end-diastolic volume (LVEDV) and microvascular obstruction (MVO) were slightly larger in the hCD16^+^ Ms group (Table 1).

**Table 1:** Baseline characteristics.

Variable	hCD16 + Ms (*n* = 11)	Control (*n* = 12)	*p*-value
Baseline weight (kg)	63.1 ± .2.7	63.2 ± 3.1.	0.92
Duration coronary occlusion (min)	60	60	na
Treated Vt/Vf during MI	6 (54.5%)	5 (41.7%)	0.84
Successful CPR during Mi	0 (0%)	1 (8.3%)	1.00
Successful DC cardioversion during Mi	3 (27.3%)	5 (41.7%)	0.78
Treated Vt/Vf during injections	0 (0%)	1 (8.3%)	1.00
DC cardioversion during injections	(0%)	1 (8.3%)	1.00
Hypotension/bradycardia During recovery	0 (0%)	0 (0%)	na
Median hCD16 + Ms number (million)	** **	** **	–
Median hCD16 + Ms viability (%)	87	–	–
Key baseline CMR outcomes			
LV scar before injections (gr)	25.4 ± 8.2	18.8 ± 51	0.03
LVEF before injections (%)	45.5 ± 6.3	44.3 ± 55	0.61
LVEDV before injections (ml)	1701 ± 28.3	1554 ± 211	0.18
Median MVO (gr)	3.00 (2.00, 7.00)	1.00 (0.00, 2.50)	0.06

VT/VF, ventricular tachycardia/ventricular fibrillation; MI, myocardial infarction; CPR, cardiopulmonary resuscitation; DC, direct current; CMR, cardiac magnetic resonance; LV, left ventricle; LVEF, left ventricular ejection fraction; LVEDV, left ventricular end-diastolic volume; MVO, microvascular obstruction.

*Feasibility and safety outcome*. Human CD16^+^ Ms were successfully extracted from leucocyte cones according to the technology shown in Supplementary Figure S2. The antigenic profile was confirmed via flow cytometry. Median monocyte number was 48.75 million, while average cell viability was 87%. Feasibility/safety measures related to coronary occlusion, VT/VF during MI or during injections and vital parameters are reported in Table 1. All 23 recruited animals survived the MI and injection procedures and reached the final 30-day post-injection time point in good health, with no evidence of infection or other clinically unacceptable complications such as malignant VT/VF associated with the treatment delivered. The overall median weight at 30-day post-injections was 82 kg. The average weight gain was 16.5 kg in keeping with normal expectations and animal well-being, with no difference between groups. Serial full blood counts showed no differences between groups over time (Supplementary Table S2). CMR safety measures at 30-day post-injections showed no evidence of intramural growth/cancer, haematoma, excessive scarring, thrombosis, infection/abscess or adverse LV remodelling associated with the use of hCD16^+^ Ms (Supplementary Table S1). This was confirmed by safety macroscopy evaluation of the whole heart after culling with all these key outcome measures being classified as absent before proceeding to heart sectioning for histology.

### Efficacy outcome measures

#### Co-primary CMR outcomes

##### LV scar size

LV scar size decreased in both groups vs. baseline to 10.8 ± 3.4gr and 12.4 ± 3.9gr in the hCD16^+^ Ms and control groups, respectively (*p* = 0.69) (Figure 3A; Supplementary Table S1). However, compared to before injections, LV scar size dropped by 14.5gr at the 30-day time point in the hCD16^+^ Ms group (from 25.4 ± 8.2 to 10.8 ± 3.4gr; 55%) vs. 6.4gr in the control group (from 18.8 ± 5.1 to 12.4 ± 3.9gr; 30%) (*p* = 0.015; Table 1; Figure 3B). Despite this baseline difference, LV scar size at 30-day post-injections was 1.6gr smaller in the hCD16^+^ Ms vs. control (10.8 ± 3.4gr vs. 12.4 ± 3.9gr, respectively) (Figure 3A). Representative CMR LV scar scans from a hCD16^+^ Ms experiment before injections and 30-day after injections are shown in Figure 3C-D. *LVEF:* At 30-day post-injections, LVEF was 40.7 ± 1.6% and 41.9 ± 1.6% in the hCD16^+^ Ms group and control group, respectively (*p* = 0.58) (see Supplementary Table S1).

#### Effect of hCd16^+^ Ms count and viability on LV scar size and LVEF

The h-CD16 + Ms group was split by medians (cell viability ≥87%: *n* = 5 vs. <87%: *n* = 6; cell count ≥48.75 million: *n* = 5 vs. <48.75 million: *n* = 6). The predefined sub-analysis showed that the use of a higher hCD16 + Ms dose was associated with 2.8% higher LVEF and 1.9 gr smaller LV scar compared to the lower cell dose (Supplementary Table S3).

### Secondary CMR outcome measures

Secondary CMR outcome measures are reported in Supplementary Table S1. At 30-day post-injections, LVEDV was 185 ml (161, 190) vs. 162 ml (148, 175) in the hCD16^+^ Ms and control groups, respectively (*p* = 0.18). MVO was 0.18 ± 0.4gr vs. 0.0 ± 0gr in the hCD16^+^ Ms and control groups, respectively (*p* = 0.15).

#### Histological outcome

**Figure 4:**
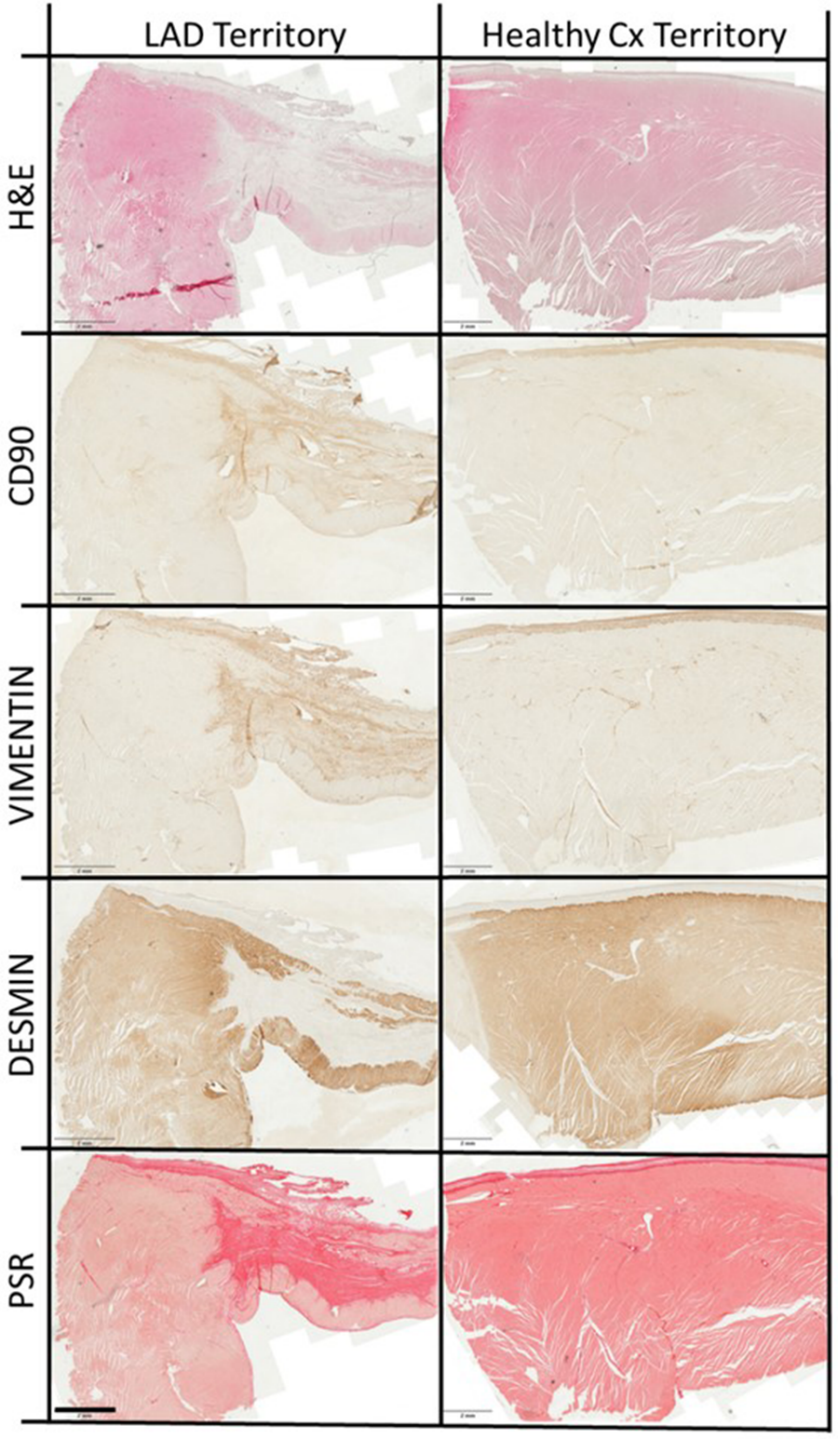
Representative myocardial staining. Representative overview images of left anterior descending (LAD) artery territory encompassing scar, border zone and transition zone, and healthy circumflex (Cx) artery territory, stained with haematoxylin and eosin (H&E), antibodies to CD90, vimentin and desmin (proteins = brown) and picrosirius red (PSR; collage*n* = red). Scale bars are 2 mm.

Safety macroscopy examinations of the hearts 30 days after injections excluded the occurrence of intramural growth/cancer, haematoma, excessive scarring, thrombosis or adverse LV remodelling associated with the use of hCD16 + Ms. In addition, histological evaluations of the liver, lung and spleen at the same time point were all normal (data not shown). Representative images of myocardial tissue stained for key markers are shown in Figure 4. Higher magnification images for each region are shown in Supplementary Figure S2. The primary antibodies used are shown in Supplementary Table S4.

### Neo-angiogenesis

**Figure 5:**
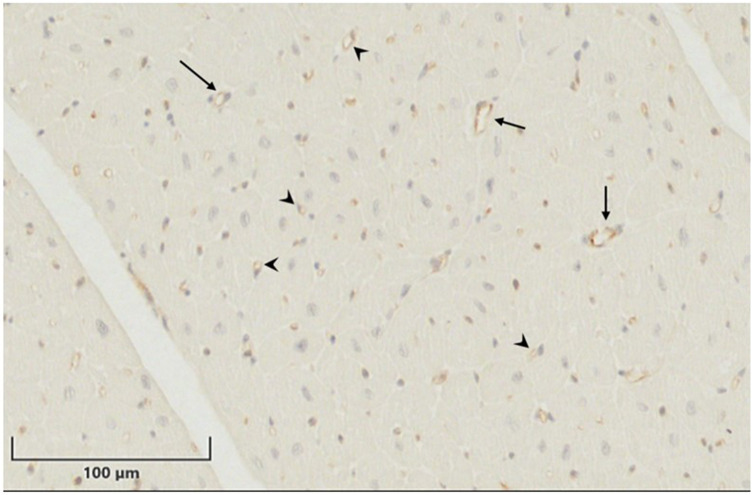
Cd31 staining to highlight capillaries and arterioles. Representative image of heart tissue stained with antibody to CD31 to highlight capillaries (arrowheads) and arterioles (arrows) manually counted in the tissue. Scale bar = 100 μm.

Results for anti-CD31 staining for separate S, BZ and TZ and H regions between groups are shown in Table 2 and Figure 5. The use of hCD16^+^ Ms increased the number of capillaries in S, BZ, TZ and H regions and of arterioles in S and TZ regions vs. controls (all *p* < 0.05). The number of capillaries and arterioles varied across regions, groups and time-points (all *p* < 0.001). Neo-angiogenesis analysed as cumulative LAD infarct region (S + BZ + TZ) and healthy CX region vs. control group showed a similar effect of hCD16^+^ Ms (see Supplementary Table S5).

**Table 2:** Neo-angiogenesis for separate S, BZ and TZ and H between groups.

		No. of vessels/mm^2^		
	Tissue region	Capillaries	Arterioles	Capillaries + Arterioles
Control	Scar	91.7 ± 25.4^$$^	38.0 ± 7.5^$$^	129.6 ± 31.7^$$^
	Border zone	122.1 ± 35.9^$$^	12.7 ± 2.2^$$^	134.8 ± 37.6^$$^
	Transition zone	80.5 ± 22.7^$$^	8.8 ± 2.9^$$^	89.4 ± 23.3^$$^
	Healthy	200.6 ± 50.9^$$^	20.2 ± 4.7^$$^	220.8 ± 53.2^$$^
hCD16 + Ms	Scar	220.4 ± 77.7*^,^^$$^	43.7 ± 7.4*^,^^$$^	264.0 ± 74.4*^,^^$$^
	Border zone	150.6 ± 51.9*^,^^$$^	14.0 ± 2.8^$$^	164.6 ± 52.7*^,^^$$^
	Transition zone	164.0 ± 53.1*^,^^$$^	17.0 ± 3.2*^,^^$$^	181.0 ± 55.7*^,^^$$^
	Healthy	318.8 ± 99.4*^,^^$$^	21.2 ± 3.8^$$^	340.0 ± 100.0*^,^^$$^

Number of capillaries and arterioles per mm^2^ of left anterior descending (LAD) territory and circumflex (CX) artery healthy regions in control vs hCD16 + Ms treated tissue (*n* = 8 pigs per group).

* *p < 0.05: effect of hCD16 + Ms treatment vs controls.*

^$$^ *p < 0.001: effect of region (both by univariate analysis of variance)*.

**Figure 6:**
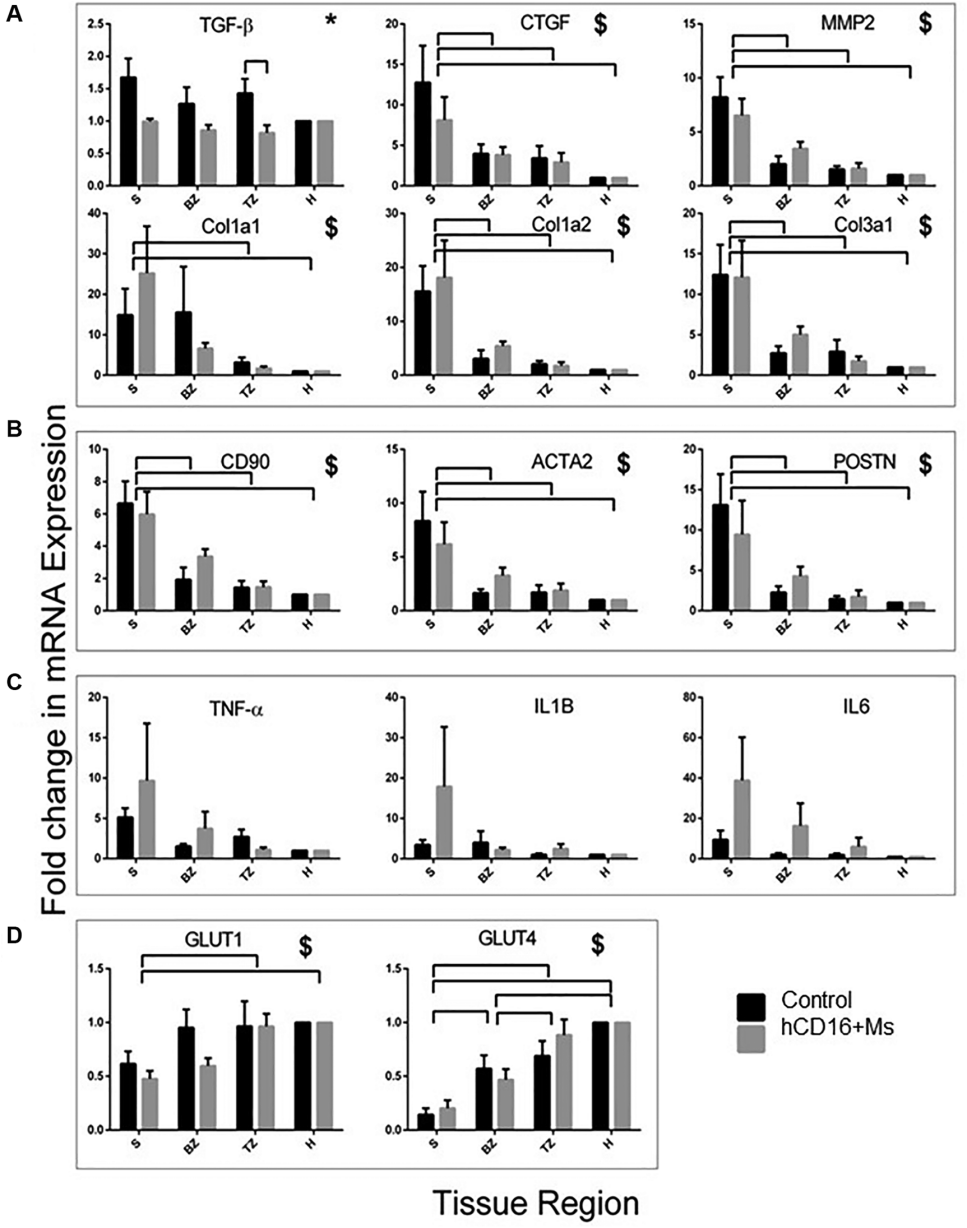
mRNA expression of genes at 30-day post-injections. mRNA expression of genes associated with fibrosis (**A**), myofibroblast function (**B**), inflammation (**C**) and heart function (**D**) in infarcted regions. Data are shown as fold-change from healthy Cx territory regions. *Significant effect of treatment. ^$^Significant effect of region. Bars over charts indicate significant differences between groups and regions. S, BZ, TZ and H indicate scar, border zone, transition zone and healthy heart regions, respectively.

Expression of mRNAs for TGF-β, CTGF(CCN2), MMP2, Col 1a1, Col 1a2and Col 3a1 across S, BZ, TZ and H regions is shown in Figure 6A. Primer sequences used are shown in Supplementary Table S6. The use of hCD16^+^ Ms reduced the levels of pro-fibrotic factor TGF-β in the TZ region (*p* < 0.01; Figure 6A) and the whole infarct (S + BZ + TZ) (Supplementary Figure S4A). No other differences related to hCD16^+^ Ms were noted.

Staining for collagen, a contributing factor to myocardial fibrosis, across S, BZ, TZ and H regions is shown in Table 3, Figure 4 and Supplementary Figure S3. Collagen levels were higher in region S and gradually reduced moving away from S through BZ, TZ and H regions. There was an effect related to hCD16 + Ms.

**Table 3:** Protein expression by staining.

		Stain expression per tissue area (%)			
	Tissue region	CD90*^,^^$$^^,^^#^	Vimentin*^,^^$$^	Desmin^$$^	Collagen^$$^
Control	Scar	11.7 ± 3.1	21.2 ± 3.0	1.4 ± 0.3	39.3 ± 9.6
	Border zone	1.0 ± 0.3	2.9 ± 0.6	75.3 ± 3.9	6.7 ± 2.4
	Transition zone	0.8 ± 0.5	2.5 ± 0.7	70.8 ± 3.1	5.8 ± 2.3
	Healthy	0.6 ± 0.1	2.1 ± 0.4	62.0 ± 4.9	4.1 ± 1.1
hCD16 + Ms	Scar	31.1 ± 9.1	41.7 ± 11.2	3.1 ± 1.4	49.6 ± 5.6
	Border zone	2.8 ± 1.3	6.2 ± 1.6	69.4 ± 8.4	8.4 ± 2.4
	Transition zone	3.5 ± 1.9	5.9 ± 1.8	63.8 ± 10.1	7.0 ± 2.2
	Healthy	1.0 ± 0.4	2.1 ± 0.4	64.5 ± 7.2	6.7 ± 2.3

Protein expression in Control vs. hCD16 + Ms hearts (*n* = 8 per group) as percentage of tissue area, in predefined regions of the infarcted heart.

* *p* < 0.05: effect of treatment by univariate analysis of variance.

^$$^ *p* < 0.001: effect of region by univariate analysis of variance.

^#^ *p* < 0.05: interaction between region and treatment by univariate analysis of variance.

### Fibroblasts and myofibroblasts

The presence of fibroblasts and myofibroblasts across S, BZ, TZ and H regions and groups was assessed by staining for anti-CD90 and anti-vimentin, respectively (Table 3; Figure 4; Supplementary Figure S3) and by mRNA expression of CD90, ACTA2 and POSTN (Figure 6B). The use of hCD16^+^ Ms was associated with increased staining for fibroblasts and myofibroblasts between groups and across S, BZ and TZ regions (both *p* < 0.05). Evaluations of mRNA expression markers did not show differences between groups.

### Cardiomyocyte function

The level of cardiomyocyte function across S, BZ, TZ and H regions and groups was assessed by staining for desmin (Table 3, Figure 4; Supplementary Figure S3) and by mRNA expression GLUT-1 and GLUT-4 (Figure 6D). Desmin was absent in S regions but was quite intense in BZ regions in both groups, with no difference associated with the use of hCD16^+^ Ms. mRNA expression of GLUT-1 and GLUT-4 increased gradually from S to H regions with no difference associated with the use of hCD16^+^ Ms.

### Tissue inflammation

Tissue inflammation across S, BZ, TZ and H regions and groups was assessed by mRNA expression TNF-α, IL-1B and IL-6 (Figure 6C). Tissue levels of IL-6 appeared to be higher in the hCD16^+^ Ms group although this was not quite significant (*p* = 0.06). No other differences were noted.

## Discussion

This preclinical trial has tested the feasibility, safety and efficacy of intramyocardial modulation with hCD16^+^ Ms delivered via intramyocardial injections early after acute MI. A rigorous approach was used including clinical grade manufacturing of hCD16^+^ Ms, randomization and blinding, longitudinal *in vivo* imaging at clinical standards via 3 T CMR, clinical grade immunosuppression and adhesions to ARRIVE guidance (25).

Feasibility and safety of hCD16 + Ms therapy were confirmed with 100% survival of all recruited and injected animals to the predefined endpoint, with normal vital parameters and serial blood cell counts, no infections, normal nutrition and well-being over time. Indeed, the average weight gain of 16.5 kg at the final time point was within expected reference intervals with no difference between groups. Additional safety outcome measures, based on *in vivo* CMR and final histopathology, excluded the presence of LV intramural growth/cancer, haematoma, excessive scarring, thrombosis, infection/abscess or adverse LV remodelling associated with the use of hCD16^+^ Ms.

The use of clinical grade immunosuppression was agreed on balance at the study design stage to prevent rejection/death of human monocytes given the xenotransplantation approach, although we appreciated that this approach might taper down the reparative capacity of the injected monocytes. Others have used immunosuppression when testing other human cells in large animals (28) reporting at times evidence of beneficial effects. The immunosuppression regimen used in this study was selected against another clinically approved regimen in a pilot study completed in the same MI model before starting this trial. This pilot study confirmed that the immunosuppression regimen used in this trial was more effective in tapering down the porcine myocardial tissue immune response without triggering infection (data not shown but available). It might be argued that the use of immunosuppression might have limited the effectiveness of the human monocytes used in this porcine trial, and therefore that using these cells on pure autologous clinical grounds, e.g., autologous hCD16^+^ Ms being used in MI patients, could lead to larger effect sizes. While these speculations make sense clinically, they could be probed further only in a future human study.

The use of hCD16^+^ Ms derived from leucocyte cones via clinical grade manufacturing provided consistent cell count, purity and viability. Of note, a higher cell dose could be easily obtained simply by expanding the leucocyte cone base, with the potential to enhance future clinical application of myocardial immunomodulation within 24–48 h from acute MI. The use of this monocyte subtype was prompted by our previous preliminary work suggesting proangiogenic activity, 10-fold higher circulating levels in critical limb ischemia patients vs. controls and resolution of limb ischemia with normalization of circulating levels (20). With regard to mechanistic pathways involved, others have also suggested that CD16^+^ Ms are associated with neo-angiogenesis (21) and that monocytes (and macrophages) play pivotal roles in cardiac healing after MI (9, 29, 30), possibly by regulating the cardiomyocytes to non-cardiomyocytes interface through their phagocytic and paracrine activities (9). Monocyte phagocytic activity is of interest in acute MI as it appears to help the removal of post-MI debris and to modulate the resolution of neutrophil recruitment toward healing, connective tissue remodelling and fibrosis (9, 29–31). It has also been reported that CD16^+^ monocytes express high levels of CX3CR1 and low levels of chemokine receptors in the infarcted myocardium, where their presence peak at about 3–5 days post-MI becoming the predominant monocyte subtype (22). In addition, they can modulate both inflammatory and reparative phases in the infarcted myocardium by releasing anti-inflammatory cytokines and growth factors to stimulate cell proliferation, angiogenesis and ECM production (23, 24). Furthermore, they may produce specialized pro-resolving lipid mediators that may facilitate the recruitment of additional monocytes, tissue repair and return to homeostasis (32).

The use of hCD16 + Ms was associated with a 55% reduction in LV scar size compared to the pre-injection time point vs. 30% reduction observed in the control group (*p* = 0.015). This marked beneficial effect on LV scar was achieved despite by chance the MI scar size at CMR was larger in the hCD16 + Ms group at the baseline pre-injections time point vs. control group (*p* = 0.03) along with larger LV volume and more MVO. However, the use of hCD16^+^ Ms at the observed dose of 48.7M did not show a difference in LVEF at 30-day post-injections. Of note, the trial was powered on the assumption of detecting a 7.2% difference in LVEF, which in retrospect might have been an optimistic assumption. No prospective consideration was given to cell dose during sample size calculation, a part of a prespecified sub-analysis within the hCD16^+^ Ms group aimed at probing associations between cell dose (and cell viability) and LVEF and LV scar size. This sub-analysis showed 2.8% higher LVEF coupled with 1.9gr less LV scar in the subgroup of animals receiving ≥48.75 million (*n* = 5) vs. those receiving <48.75 million (*n* = 6). This secondary finding might be not trivial and may warrant an expanded preclinical trial focusing on higher cell doses. It might be argued that not all the hCD16^+^ Ms injected might have been retained. However, we used a well-established method of direct intramyocardial injections via a small left thoracotomy which is clinically feasible, has been used in the past in humans and animals (33–35) and has shown to achieve a higher amount of cell retention compared to intracoronary injection or IV infusion routes (36).

We also note that the decline in LVEF observed 5 days post-MI and before injections across the groups was in the moderate range (LVEF dropped only approx. 45%) as opposed to a more severe LV function impairment (LVEF <35%). According to others, a starting degree of LV moderate impairment (around 45% makes it more difficult to achieve 5%–8% gains in LVEF in absolute terms when testing new therapies) (27, 37).

The use of hCD16 + Ms was associated with higher neo-angiogenesis, including both capillaries and arterioles, in the infarcted myocardial territory. This finding is novel for the MI clinical setting as similar results have been observed previously in the context of limb ischemia (20) and other non-cardiac conditions (21). The increase of both capillaries and arterioles could explain the observed reduced LV scar size. Arterioles tend to regulate tissue blood flow while neo-capillaries increase blood flow in myocardial ischemic areas at risk; hence, this higher neo-angiogenesis might have contributed to saving cardiomyocytes at risk in our model. This finding in pig might be relevant to future human studies as we have recently reported that myocardial microcirculation in pig is structurally and functionally similar to myocardial microvasculature in humans (38).

The reduced expression of pro-fibrotic factor TGF-β in the hCD16 + Ms group might have contributed to the reduction in LV scar size seen in the hCD16 + Ms group. Concomitantly, the larger amount of tissue anti-vimentin in the hCD16 + Ms group indicates beneficial healing/function with higher levels of myofibroblasts, in keeping with the notion that myofibroblasts may effectively repair/remodel the interstitium following MI (39–41). The use of hCD16^+^ Ms was also associated with a trend of higher IL-6 tissue levels vs. controls at 30-day post-injections (*p* = 0.06). Accordingly, loss of IL-6 has been associated by others with ventricular dysfunction, fibrosis, reduced capillary density and worse myocardial reparative features (42). However, others have identified detrimental effects associated with increased IL-6 myocardial levels early after MI (8).

It might be argued that the sample size of our porcine trial was too small. However, the sample size was in line with the predefined calculation reported in Methods. Of note, the used sample size is in keeping with previous meta-analyses from 52 large-animal studies indicating that, in order to obtain a power of at least 80% in a two-sided two-sample *t*-test with an alpha of 0.05, 11 animals are needed in each group to detect a clinically meaningful difference in LV ejection fraction (43). In addition, it exceeded the sample size of most of the 82 studies in a total of 1,415 large animals reported in a very large systematic review (28). A limitation of this study might be that the immunosuppression regimen, commenced on Day 3 post-MI, could have impacted the early myocardial healing with possible confounding. However, similar immunosuppression was undertaken in both groups, hence any effect observed in the hCD16^+^ Ms group should be genuine. Another limitation of this study might be that the predefined endpoint of 30 days after injections represented a relatively too short follow-up time period. A longer period of follow-up might have helped assess the long-term impact of the proposed intramyocardial immunomodulation of key outcome measures including LV pathological remodelling, which tends to occur over a longer time period in patients with ischemic heart failure.

In conclusion, this preclinical trial suggests that the use of hCD16^+^ Ms to treat acute MI is feasible and safe and it is associated with reduced LV scar size, increased tissue levels of neo-angiogenesis and myofibroblasts and reduced pro-fibrotic TGF-β at 30 days despite immunosuppression. While no effect was observed on LVEF, a predefined sub-analysis within the hCD16 + Ms group showed a trend for better effect size on LVEF and scar when a higher cell dose was used. More studies are warranted to probe the efficacy of the proposed treatment.

## Data Availability

The raw data supporting the conclusions of this article will be made available by the authors, without undue reservation.
