# Supplementary material for: Intramyocardial immunomodulation with human CD16+ monocytes to treat myocardial infarction in pig: a blind randomized preclinical trial
Source: Front Cardiovasc Med. 2024 Aug 7;11:1427023. doi: 10.3389/fcvm.2024.1427023 (PMC11335517; doi:10.3389/fcvm.2024.1427023)
Supplement: Supplementary file 1 [file Datasheet1.docx]

## Supplementary file

**Extended Materials, Methods and Results.**

*Porcine MI procedure and delivery of* hCD16^+^Ms

Female Large White swine were used (weight 55-60 kg). The MI procedure was as previously reported (24). Briefly, aspirin 300mg daily was administered daily with food from 5 days before MI and until termination (Aspirin Tablets (acetylsalicylic acid 75 mg; Accord, Barnstaple, UK). On the MI day, under general anaesthesia (GA), full monitoring, and IV heparin (Heparin Sodium 5,000 I.U./mL preservative free; Wockhardt UK Ltd., Wrexham, UK) administered to achieve an activated clotting time (ACT) > 300 sec, animals had their left anterior descending (LAD) artery wired percutaneously via a 6F sheath inserted in the right carotid artery. The LAD was occluded for 60min distal to the first diagonal branch using a 1:1-1.25 sized angioplasty balloon (Ryujin balloon, Terumo Japan) under fluoroscopic guidance (Siemens Artis Zee floor mounted). Amiodarone infusion (300mg over 90min; Amiodarone hydrochloride 50 mg/mL; hameln pharma ltd, Hameln, Germany) was started before coronary occlusion to prevent ventricular fibrillation (VF). Additional amiodarone bolus (150mg) and DC cardioversion were used to treat VF during ischemia. On completion of 60min, the coronary balloon was deflated (with angiographic check) and removed. Animals were recovered and vital parameters monitored for 45-60min before return to the maintenance area. Daily clinical grade immunosuppression was started 3 days after MI and included cyclosporine 15mg/kg/day + methylprednisolone 2mg/kg/day till termination. In-vivo characterisation of MI size and LV function occurred 5 days after MI under GA via baseline cardiac magnetic resonance (CMR) imaging. Following CMR imaging, animals were randomized according to a predefined sequence to receive blindly either hCD16^+^Ms suspended in saline (hCD16^+^Ms group) or saline alone (Control group) via 10 micro-injections (0.3mL each) targeting LV scar and border zone via mini-thoracotomy. On completion, the small thoracotomy was closed in layers and injected with local anaesthetic combined with subsequent intravenous paracetamol (Paracetamol 10mg/mL solution for infusion; Fresenius Kabi Limited, Runcorn, UK), buprenorphine and transdermal fentanyl patches (Sandoz, Novartis Pharmaceuticals Limited, UK) for postoperative pain relief. 30-day after injections, animals underwent a final GA, repeat CMR followed by termination and tissue sampling.

*Macroscopy and myocardial histological staining*

Whole heart macroscopy was undertaken soon after termination by blind pathologists reporting on presence/absence of LV intramural growth/cancer, infection/abscess, and/or intramural haematoma/thrombosis. Each LV territory was assessed against the LV 17-segments schematic, also used for recording on LV injection sites at surgery (see Figure S1). Macroscopic abnormal findings would be subjected to histology only if classified as present.

With regard to routine myocardial histology, a total of 8 hearts randomly selected in each group were used. Tissue evaluation consisted of a combination of histochemical staining and mRNA expression for each area, with focus on: neo-angiogenesis, fibrosis, myofibroblast function, cardiomyocyte function, and inflammation.

*Myocardial tissue staining:* Histological staining was performed blindly on tissue sections following standard protocols for markers of neoangiogenesis (anti-CD31/PECAM-1; platelet endothelial cell adhesion molecule/), fibrosis (picrosirius red for collagen), myofibroblasts (anti-vimentin and anti-CD90 (Thy1)), and heart function (anti-desmin as a marker of functional cardiomyocytes). All stained tissue sections were imaged with a Precipoint O8 Slidescanner using a 20x objective, with the exception of CD31 stained tissue that was imaged at higher magnification (40x). To quantify neo-angiogenesis, anti-CD31 (a marker of endothelial cells) was assessed across the average of five images per heart region, with manual counting of capillaries (CD31^+^ staining with one associated nuclei) and arterioles (CD31^+^ with two or more nuclei; Figure S1) with data reported as the number of vessels/mm^2^ tissue. Remaining histological analysis was performed on data from three regions per area, and the percentage of staining per tissue area determined using colour deconvolution in Fiji ImageJ (Java 1.8.0 64-bit). 2-way ANOVA was used to determine significant effects of treatment (Control vs Stem Cell) and region.

*mRNA Expression:* this was determined using 384-well quantitative PCR for markers of myocardial fibrosis (TGF-β, CTGF, MMP2, Col1a1, Col1a2, Col3a1), myofibroblasts (CD90, ACTA2, POSTN), cardiomyocyte function (GLUT1, GLUT4) and inflammation (TNFα, IL-1B, IL-6). All mRNA levels were normalised to the housekeeping genes GUSB, PPIA and GAPDH. Expression was calculated using the 2^–ddCt method, and expression for each region within the LAD territory was compared as fold-change to expression within healthy Cx territory regions for each heart. Additional analysis was also performed comparing healthy versus infarcted regions combined. 2-way ANOVA was used to determine significant effects of treatment (Control vs Stem Cell) and region, and a post hoc Tukey HSD test carried out to observe differences between individual regions.

Tissue sections were dewaxed and rehydrated before undergoing subsequent staining. For immunohistochemistry, heat-mediated antigen retrieval was performed using 10mM citrate buffer (pH 6.0). Sections were incubated in Bloxall (Vector) to inhibit endogenous peroxidase, followed by Trident Universal Protein Blocking Reagent (animal serum free) (GeneTex). Primary antibodies (Table S2) were added and incubated overnight at 4°C. Following removal of primary antibody, biotinylated secondary antibodies were added (one hour at room temperature (RT)). Extravidin peroxidase (Sigma-Aldrich) was added followed by 3,3-diaminobenzidene (DAB; Sigma-Aldrich), before counterstaining with haematoxylin and mounting. For collagen staining, following tissue rehydration, sections were stained in picrosirius red (PSR) solution (90minutes, RT), rinsed in hydrochloric acid (0.01N) and then mounted for imaging.

**Table S1**. CMR outcomes measures before injections and at 30-day after injections

|  | hCD16+Ms (n=11) | Control (n=12) | **p-value^1^** |
| --- | --- | --- | --- |
| **CMR outcomes before injections** |  |  |  |
| LV Scar mass(gr) | 27 (18, 32) | 20 (17, 21) | 0.11 |
| LVEF (%) | 45.0 (40.5, 47.0) | 44.0 (41.5, 48.0) | 0.65 |
| LVEDV (ml) | 172 (162, 189) | 155 (138, 172) | 0.17 |
| LVEDV indexed to weight(ml/kg) | 103 (94, 112) | 97 (84, 104) | 0.32 |
| LVESV (ml) | 90 (84, 104) | 82 (78, 96) | 0.26 |
| LVESV indexed to weight (ml/kg) | 53 (50, 62) | 51 (48, 56) | 0.51 |
| LV mass (grams) | 110 (106, 118) | 101 (95, 112) | 0.12 |
| Indexed LV mass (gr/kg) | 64 (62, 70) | 62 (58, 68) | 0.31 |
| SV | 83 (61, 91) | 69 (65, 78) | 0.18 |
| SV indexed to weight | 49 (37, 54) | 44 (39, 48) | 0.37 |
| CO (L/min) | 8.90 (7.20, 9.65) | 7.20 (6.65, 8.40) | 0.082 |
| MVO mass (gr)* | 3.00 (2.00, 7.00) | 1.00 (0.00, 2.50) | 0.060 |
| **CMR outcomes at 30-day post-injections** |  |  |  |
| LV Scar mass(gr) | 9.0 (9.0, 12.5) | 14.0 (8.0, 16.0) | 0.49 |
| LVEF (%) | 40.1 (37.0, 42.0) | 41.2 (38.0, 44.0) | 0.62 |
| LVEDV (ml) | 185 (161, 190) | 162 (148, 175) | 0.18 |
| LVEDV indexed to weight (ml/kg) | 100 (89, 107) | 90 (82, 94) | 0.12 |
| LVESV (ml) | 115 (100, 119) | 98 (82, 107) | 0.069 |
| LVESV indexed to weight (ml/kg) | 61 (54, 64) | 55 (44, 56) | 0.055 |
| LV mass (gr) | 115 (112, 128) | 117 (110, 126) | 0.67 |
| LV mass indexed to weight (gr/kg) | 63 (61, 70) | 67 (59, 74) | >0.99 |
| SV | 71 (62, 82) | 68 (60, 76) | 0.35 |
| SV indexed to weight | 40.0 (34.0, 44.5) | 35.5 (33.0, 42.0) | 0.56 |
| CO (L/min) | 8.10 (7.75, 8.90) | 8.55 (7.52, 9.25) | 0.88 |
| MVO mass (gr)* | 0.18(0.4) | 0(0) | 0.15 |
| LV intramural growth/cancer | 0 | 0 | NA |
| LV intramural infection/abscess | 0 | 0 | NA |
| LV intramural haematoma/thrombosis | 0 | 0 | NA |
| *All data are reported as Median (IQR). *: data are reported as mean (SD). ^1^Wilcoxon rank sum exact test. LV; Left Ventricle; LVEF: Left Ventricular Ejection Fraction; LVEDV: Left Ventricular End Diastolic Volume; LVESV: Left Ventricular End Systolic Volume; SV: Stroke Volume; CO: Cardiac Output; MVO: Microvascular obstruction*. | | | |

**Table S2:** Serial Full blood count overtime in both groups

| Variable | hCD16+Ms (n=11) | Control (n=12) | p-value |
| --- | --- | --- | --- |
| Baseline before MI |  |  |  |
| WBC before injection (x10^9/l) | 17.39 ± 2.79 | 16.73 ± 3.37 | 0.61 |
| Hb before injection (g/dL) | 9.48 ± 0.76 | 9.39 ± 0.6 | 0.76 |
| Neutrophils before injection (x10^9/l) | 5.55 ± 1.63 | 4.37 ± 1.34 | 0.13 |
| 5-day post MI and before injections |  |  |  |
| WBC at injection (x10^9/l) | 19.12 ± 6.14 | 18.89 ± 5.87 | 0.93 |
| Hb at injection (g/dL) | 9.54 ± 0.85 | 9.57 ± 0.87 | 0.94 |
| Neutrophils at injection (x10^9/l) | 13.84 ± 5.23 | 12.99 ± 6.26 | 0.72 |
| 30-day after injections |  |  |  |
| WBC at termination (x10^9/l) | 16.89 ± 2.16 | 16.89 ± 3.97 | 0.99 |
| Hb at termination (g/dL) | 9.93 ± 0.9 | 9.74 ± 0.76 | 0.61 |
| Neutrophils at termination (x10^9/l) | 11.75 ± 3.1 | 12.04 ± 4.36 | 0.86 |
| *Data are presented as mean ± SD. MI: Myocardial Infarction; WBC: white blood cells; Hb: Haemoglobin.* | | | |

## Table S3: Pre-specified sub-analysis within Cell group for cell-dose and cell viability

|  | hCD16+Ms (n=11) | | Control (n=12) | Difference in means (SE) | |
| --- | --- | --- | --- | --- | --- |
| Cell viability (%) | ≥ 87(n=5) | < 87(n=6) |  | ≥ 87(n=5) | < 87(n=6) |
| LVEF before injections (%) | 45.5(2.6) | 45.6(2.8) | 43.4(1.8) |  |  |
| LVEF at 30-day (%) | 41.1(2.2) | 40.3(2.4) | 41.9(1.6) | -0.9(2.7) | -1.7(2.9) |
| Cell dose (M) | ≥ 48.7(n=5) | < 48.7(n=6) |  | ≥ 48.75(n=5) | < 48.75(n=6) |
| LVEF before injections (%) | 47.2(2.5) | 43.6(2.8) | 43.4(1.8) |  |  |
| LVEF at 30-day (%) | 42.0(2.2) | 39.2(2.3) | 41.9(1.6) | 0.1(2.7) | -2.7(2.8) |
| Cell viability (%) | ≥ 87(n=5) | < 87(n=6) |  | ≥ 87(n=5) | < 87(n=6) |
| LV scar at injections (gr) | 26.2(2.8) | 24.6(3.0) | 18.9(1.9) |  |  |
| LV scar at 30-day (gr) | 10.6(1.6) | 9.7(1.7) | 13.0(1.1) | -2.4(1.9) | -3.2(2.0) |
| Cell dose (M) | ≥ 48.7(n=5) | < 48.7(n=6) |  | ≥ 48.7(n=5) | < 48.7(n=6) |
| LV scar at injections (gr) | 25.8(2.8) | 25.0(3.0) | 18.9(1.9) |  |  |
| LV scar at 30-day (gr) | 9.3(1.5) | 11.2(1.6) | 13.0(1.1) | -3.7(1.9) | -1.8(2.0) |

*Data are presented as mean ± SD. LVEF: Left Ventricular Ejection Fraction; M; Million; LV: Left Ventricle*.

**Table S4:** Antibodies used for immune-histochemistry

| **Primary Antibody** | **Catalogue No. / Supplier** | **Working Concentration** | **Secondary Antibody** | **Catalogue No. / Supplier** | **Working Concentration** |
| --- | --- | --- | --- | --- | --- |
| Rabbit Anti-PECAM-1 / CD31 | Ab28364 / Abcam | 0.45 μg/ml | Goat anti-rabbit IgG (Whole molecule) | B7389 / Sigma | 3.5ug/ml |
| Goat Anti-Desmin | AF3844 / R&D Systems | 2 μg/ml | Rabbit anti-goat IgG (H+L) | BA-5000 / Vector | 7.5ug/ml |
| Mouse Anti-Vimentin | Ab8979 / Abcam | 5 μg/ml | Goat anti-mouse IgG (H+L) | BA-9200 / Vector | 7.5ug/ml |
| Sheep Anti-Thy1 (CD90) | AF2067 / Biotechne | 2.7 μg/ml | Rabbit anti-sheep IgG (H+L) | BA-6000 / Vector | 7.5ug/ml |
| Anti-rabbit IgG | 10500C / Invitrogen | 0.45 μg/ml | Goat anti-rabbit IgG (Whole molecule) | B7389 / Sigma | 3.5ug/ml |
| Anti-goat IgG | AB-108-C / R&D Systems | 2 ug/ml | Rabbit anti-goat IgG (H+L) | BA-5000 / Vector | 7.5ug/ml |
| Anti-mouse IgG | 10400C / Invitrogen | 5 μg/ml | Goat anti-mouse IgG (H+L) | BA-9200 / Vector | 7.5ug/ml |
| Anti-sheep IgG | 5-001-A / Biotechne | 2.7 μg/ml | Rabbit anti-sheep IgG (H+L) | BA-6000 / Vector | 7.5ug/ml |

**Table S5.** Neo-angiogenesis across cumulative S+BZ+TZ and healthy regions between groups.

|  |  | **No. of vessels /mm^2^** | | |
| --- | --- | --- | --- | --- |
|  | Tissue Region | Capillaries | Arterioles | Capillaries + Arterioles |
| **Control** | Scar / BZ / TZ | 103.3 ± 26.9 | 20.9 ± 3.9 | 124.2 ± 30.4 |
|  | Healthy | 200.6 ± 50.9 | 20.2 ± 4.7 | 220.8 ± 53.2 |
| hCD16+Ms | Scar / BZ / TZ | 178.3 ± 58.3* | 24.9 ± 3.5* | 203.2 ± 58.1* |
|  | Healthy | 318.8 ± 99.4* | 21.2 ± 3.8 | 340.0 ± 100.0* |

*Number of capillaries and arterioles per mm^2^ of cumulative left anterior descending (LAD) territory and circumflex (CX) artery healthy regions in Control vs hCD16+Ms treated tissue (n=8 pigs per group). BZ: Border Zone; TZ: Transition Zone. *p<0.05: Effect of hCD16+Ms treatment vs controls by univariate analysis of variance.*

**Table S6.** Porcine primer sequences used for quantitative PCR.

|  | **Gene** | **Description** | **Primer Sequence (5’ to 3’)** |
| --- | --- | --- | --- |
| Housekeeping Genes | GUSB | Glucuronidase Beta | F: CTACTTCAAGATGCTGATCG  R: TAACTGTTCACACAGATCAC |
|  | PPIA | Peptidylprolyl Isomerase A | F: CAAAGACAGCAGAAAACTTC  R: CATTATGGCGTGTGAAGTC |
|  | GAPDH | Glyceraldehyde 3-phosphate dehydrogenase | F: GTCGGAGTGAACGGATTTGGC  R: TTGATGGCGACAATGTCCACT |
| Fibrosis | TGF-β | Transforming growth factor Beta | F: AGAGGCTATAGAGGGTTTT  R: TTGAACCCGTTAATTTCCAC |
|  | CTGF | Connective tissue growth factor / CCN2 | F: TACCGACTGGAAGACACGTTTGG  R: CCATCCCACAGGTCTTGGAACAG |
|  | MMP2 | Matrix metalloproteinase 2 | F: CGAGATCTTCTTCTTCAAGG  R: TACACAGCATCGATCTTTTC |
|  | Col1a1 | Collagen Type I Alpha 1 Chain | F: CAAGGAGCCCGAGGCTCTGAA  R: CAGCAATACCAGGAGCGCCGTT |
|  | Col1a2 | Collagen Type I Alpha 2 Chain | F: AAGGAGTCTGCATGTCTAAG  R: TTGCCTCTTGTAAAGATTGG |
|  | Col3a1 | Collagen Type III Alpha 1 Chain | F: TTTAGACATGACGAGCTTTG  R: CCTTCAATAGCTTCCTGTTG |
| Myofibroblasts | CD90 | Cluster of Differentiation 90 / Thy-1 | F: CTCTCTTGCTAACAGTCTTG  R: AGGTTGGTGGTATTCTCATG |
|  | ACTA2 | Smooth muscle alpha-2 actin | F: AATCCTGACCTTGAAGTACC  R: GCTCATTGTAGAAAGAGTGG |
|  | POSTN | Periostin | F: GAAGGAAAGGGATCATTCAC  R: TTTACATTGCTCTCCAAACC |
| Cardiomyocyte function | GLUT1 | Glucose transporter 1 | F: GATGAAGGAGGAGTGCCG  R: CAGCACCACGGCGATGAGGAT |
|  | GLUT4 | Glucose transporter 4 | F: TAAGACAAGATGCCGTCGGG  R: GAGAAGACGGCGAGGACAAG |
| Inflammation | TNF-α | Tumor necrosis factor Alpha | F: CCCAAGGACTCAGATCATC  R: GGGAGTAGATGAGGTACAG |
|  | IL-1B | Interleukin 1 Beta | F: AAGAGAGAAGTGGTGTTCTC  R: TCTTTCCCTTGATCCCTAAG |
|  | IL-6 | Interleukin 6 | F: TCTGGGTTCAATCAGGAGACCT  R: GGTCTGGATCAGTGCTTTGGTA |


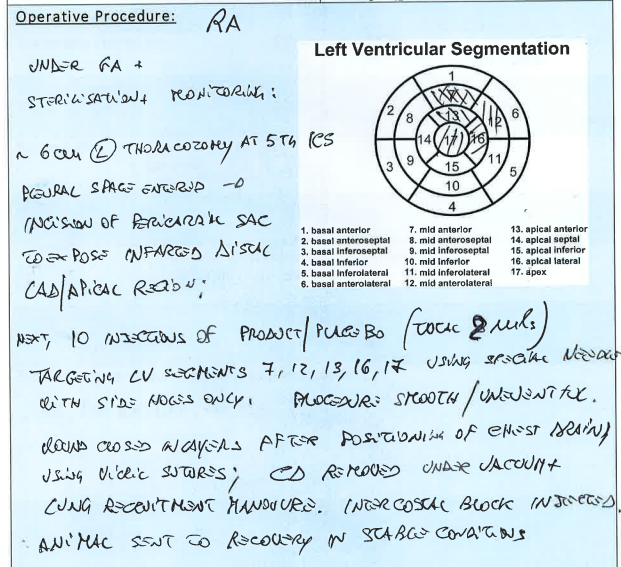
**Figure S1:** LV 17-model.

*Figure S1:* *Representative extract from operative report showing the LV 17-segment model used to record LV infarcted segments injected at surgery.*

**
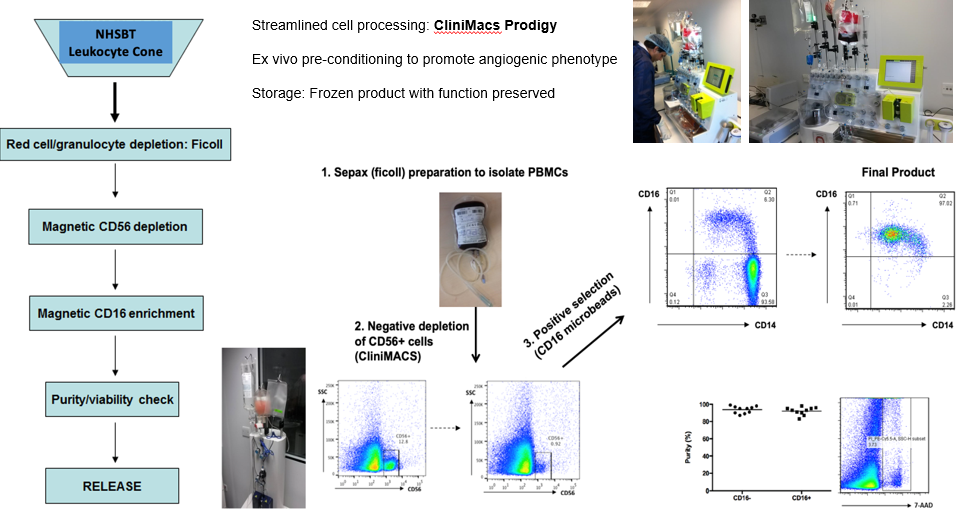
Figure S2:** Manufacturing of hCD16+ circulating monocytes

*Figure S2: h-CD16+Ms) were selected from leucocyte cones produced as by-product of the apheresis process of blood donated to the National Health Service Blood and Transplant (NHSBT) to provide samples for clinical use. The Clinimacs Prodigy (Miltenyi Biotec, Germany) system was used for clinical grade cell processing. Key manufacturing sequential steps included red cell/granulocyte depletion via Ficoll, magnetic CD56 depletion, magnetic CD16 enrichment, and final purity/viability/count checks before product storage/release at clinical standards.*

*
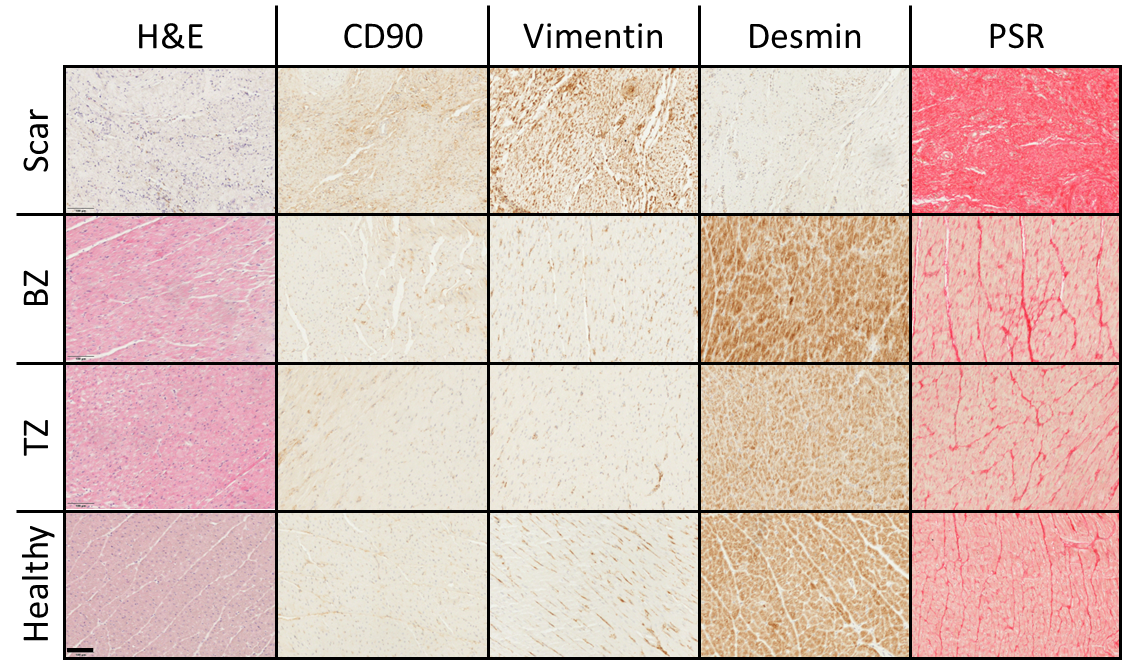
***Figure S3.** Higher magnification images for each region and used methods of staining

*Figure S3:* *Representative images of regions analysed for each zone (BZ and TZ are border and transition zones respectively). Heart tissue stained with haematoxylin and eosin (H&E), antibodies to CD90, vimentin and desmin (proteins = brown), and picrosirius red (PSR; collagen = red). All images are shown at the same scale; scale bar is 100 µm.*



**Figure S4.** mRNA expression within infarcted LAD territory

*Figure S4: mRNA expression of genes within whole left anterior descending (LAD) artery territory (S+BZ+TZ) shown as fold-change from circumplex (Cx) artery healthy region, associated with a) fibrosis, b) myofibroblast function, c) inflammation and d) heart function. Bar over TGF-β chart indicates significant differences between hCD16+Ms vs Control groups (p<0.05)*

*.*
